# National pattern of grain products consumption among Canadians in association with body weight status

**DOI:** 10.1186/s40795-017-0183-x

**Published:** 2017-08-25

**Authors:** Hassan Vatanparast, Susan Whiting, Alomgir Hossain, Naghmeh Mirhosseini, Anwar T. Merchant, Michael Szafron

**Affiliations:** 1 College of Pharmacy and Nutrition, University of Saskatchewan, 110 Science Place, Saskatoon, SK S7N 5C9 Canada; 2 University of Ottawa Heart Institute, Ottawa, Canada; 3 Department of Community Health and Epidemiology, Health Science Building, University of Saskatchewan, 107 Wiggins Road, Saskatoon, SK S7N 5E5 Canada; 4 Pure North S’Energy Foundation, 326 11 Ave SW, Calgary, AB T2R0C5 Canada; 5 Department of Epidemiology and Biostatistics, Arnold School of Public Health, University of South Carolina, 800 Sumter Street, Columbia, SC 29208 USA; 6 School of Public Health, University of Saskatchewan, 110 Science Place, Saskatoon, SK S7N 5C9 Canada; 7 College of Pharmacy and Nutrition, 104 Clinic Place (E3332 Health Sciences), Saskatoon, SK S7N 2Z4 Canada

**Keywords:** Whole-grain, Refined-grain, Adult, Obesity, National, Canadian community health survey 2004

## Abstract

**Background:**

Obesity in Canadian adults is showing upward trends. Consumption of whole-grains is one recommendation for the prevention of obesity. Despite the apparent nutritional and energy content differences between whole and refined grains, knowledge relating refined grains to weight gain in Canadian adults is scarce. The aim of this study was to assess the consumption of specific grain-based foods at the regional and national levels, and to evaluate the association between grain consumption with overweight or obesity in Canadian adults.

**Methods:**

We used the 2004 Canadian Community Health Survey data. The association between type of grain product consumed and Body Mass Index (BMI) in adults aged ≥19y was evaluated by logistic regression.

**Results:**

The mean daily intake of whole grains (86 ± 1.9 g/day) was significantly less than refined grains (276.6 ± 3.8 g/day), which was different across provinces. After adjustment for caloric needs, male consumers showed significantly lower intake of whole grains than females. Accordingly, the incidence of overweight or obesity was higher in males than in females. Also, in comparison to whole grains, the consumption of refined grains was associated with a higher risk of overweight or obesity among adults.

**Conclusion:**

Canadians’ preference was refined grain products consumption, based on 2004 Health Survey, which was significantly associated with overweight/obesity. Hence, consumption of whole grains should be more effectively promoted rather than refined grain products to prevent obesity and its complications such as cardiovascular diseases and type 2 diabetes.

**Electronic supplementary material:**

The online version of this article (doi:10.1186/s40795-017-0183-x) contains supplementary material, which is available to authorized users.

## Background

The increasing number of obese and overweight individuals in Canada has raised public attention and caused scientific debate on this issue [1, 2]. Between 1985 and 2011, the prevalence of adult obesity in Canada increased from 6.1% to 18.3%. It is predicted that by 2019, 34% and 21% of Canadian adults will be overweight and obese respectively [2–5]. The skyrocketing increase in the prevalence of obesity and its close association with major chronic diseases, also the importance of fiber in controlling body weight [6] necessitate the replacement of current diet with high percentage of refined grains, with whole-grain products.

Preliminary analyses of data from the 2004 Canadian Community Health Survey (CCHS) [7] showed that grains are a major source of energy for Canadians, contributing to 28.5% of the total energy intake of individuals with the age of over 18y [8]. In support of evidence linking whole-grain intake with a minimized risk of cardiovascular disease and reduced body weight, Canada’s Food Guide in 2007 recommended whole grains as at least half of the grain product daily consumption [9–12]. Seeds (also defined as whole grains such as Flaxseed, nuts) are not commonly consumed by Canadians, but the consumption is associated with a reduced risk of several chronic conditions including cardiovascular disease, certain forms of cancer, type 2 diabetes, and metabolic syndrome [13, 14, 11]. Many are choosing to consume refined grains as opposed to whole grains, due to their taste and appearance [15, 16]. However, the refining process removes bran, resulting in a loss of nutrients and leaving the refined grain with a higher relative starch concentration when compared to its whole grain form [17, 18]. The positive effect of whole grain products on weight loss is supported by previous research [19, 20]. However, few studies have linked refined grains to weight gain in Canadian adults [11, 21]. Further, to our knowledge, no detailed information is available on specific patterns of grain product consumption in Canada. The aim of the present study was to examine two aspects of grain product consumption in a representative sample of Canadians from the CCHS 2004 nutrition survey: 1) the consumption of specific grain-based foods and seeds by age/sex groups at national and regional levels, and 2) the association between refined grain consumption and being overweight or obese, defined by BMI, in Canadian adults.

## Methods

### Study population

The 2004 CCHS [7] sampled 35,107 individuals living in private dwellings in ten provinces [22] and the manner in which these samples were obtained adequately represents the Canadian population at provincial (only 10 provinces, excluding the territories, individuals living in institutions, correctional facilities and military service) and national levels. In the current study, the national intake of grain products was assessed using the CCHS 2.2 data on individuals aged 1 year and over (*n* = 34,818). We excluded pregnant women from this analysis. Data were collected between January 2004 and January 2005 with 98% coverage of the target population using a multi-stage cluster sampling [7].

### Provincial distribution

To avoid small sample sizes, we grouped Canadian provinces, except territories, into five regions according to their geographic locations and similar population size: 1) Atlantic Provinces (Newfoundland & Labrador, New Brunswick, Nova Scotia, and Prince Edward Island) (*n* = 6447), 2) Quebec (*n* = 4746), 3) Ontario (*n* = 10,837), 4) Prairie Provinces (Manitoba, Saskatchewan, Alberta) (*n* = 9172), and 5) British Columbia (*n* = 3616).

### Dietary exposure

Dietary intake data were collected via 24-h recall, and a second recall was conducted with one third of participants to obtain estimates of usual intakes of foods and nutrients [7]. In this study we used data from the first 24-h dietary recall which was completed by the whole CCHS 2.2 sample. We categorized participants by age/sex groupings similar to that of the Dietary Reference Intakes (DRIs) [10] by combining males and females aged 1-8y into one group, and collapsing all older adult categories into two groups: males or females >50y and males or females between 19 and 49 years. We identified all grain-based food items consumed by the participants. The consumption of various grain products was classified into three main categories: 1) whole grains, 2) refined grains, and 3) seeds (Additional file 1: Table S1). Due to the current trends in introducing whole seeds without any processing in diet, we considered seeds as a separate category. Individuals were defined as consumers of whole grain or refined grain products when more than 60% of the total daily intake of grain products was from whole grains or refined grains.

Canada’s Food Guide (2007) [10] recommends a daily consumption of Grain Products in which at least 50% of grain product choices are whole grain. The Canada’s Food Guide recommends varying amounts of grain products based on age and sex, ranging between 3 servings per day for 2–3 year olds and 8 servings per day for men 19–50 y. Examples of one serving of Grain Products could be 1 slice of bread (35 g), or ½ cup (74 g) cooked pasta, or ½ cup (93 g) cooked rice, or 30 g cold cereal.

### Data collection

Data on demographics, socio-economic status, physical activity, and food security status were collected by interview. BMI (kg/m^2^) was calculated from the measured weight (kg) and height (m). Socio-economic status (using household income and including; lower, lower middle, upper middle and high), leisure time physical activity (categorized into different levels of activity including; active, moderate, inactive), and BMI [categorized into normal (<25 kg/m^2^), overweight (25–29.9 kg/m^2^) and obese (≥30 kg/m^2^)] were compared between males and females within each group of consumers (i.e. whole grain or refined grain consumers).

### Statistical analyses

Analyses were run using the software programs *SAS 9.1*, and *STATA SE 10*. Statistical differences in intakes of grain product categories (whole grain and refined grain and seeds) between age/sex groups and/or geographic regions were evaluated by comparing confidence intervals and *p*-values. Characteristics of whole-grain and refined-grain consumers in terms of socio-economic status, leisure time physical activity, and BMI were used to compare differences between males and females. We were interested in sex-specific patterns of intake and its association with outcomes because dietary habits are different between men and women, and women are more health conscious [23].

Logistic regression was used to evaluate the association between grain product intake (whole grain users vs. refined grain users as a categorical variable) and BMI status. There was less than 3% with low BMI (<18.5 kg/m^2^) and the few cases were excluded from further analysis. Therfore, BMI was used as a categorical independent variable, and normal was a reference category in logistic regression. Possible confounders including energy intake, income-related food security status, education, area of residence, marital status, smoking, physical activity, and country of origin (Canadians-born vs. non-Canadian born) were all included in adjusted models. Due to the complex survey design, data were weighted and bootstrapped to obtain estimates that could be generalized to national and regional levels. Weighting provided unbiased estimates of population quantities, and bootstrapping allowed for estimation of standard errors, coefficients of variation and confidence intervals. One-Way ANOVA tests were used to compare different groups of grain intakes. Two-Way ANOVA tests were applied to look into the effect of both grain intake and gender/age groups. Comparison between categorical groups was done using Pearson Chi-square test. Alpha was set at 0.05.

## Results

### Assessment of grain intake

#### Intake according to grain categories and region

In Canada, the mean daily intake of all grain products was 308.7 ± 3.5 g/day. Mean intakes of different grain products including; whole grains, refined grains and seeds at national level and by specified regions are shown in Table 1. Comparison of the national daily intake of grains showed that the mean intake of whole grains was much less than that of refined grains (86 ± 1.9 g/day versus 276.6 ± 3.8 g/day, respectively), which follows the same type in different regions.

**Table 1:** Mean intakes of categories of grain products by region by Canadians 1 y and over

		All grain products		Whole grains^*^		Refined grains^#^		Seeds^^^	
N		Mean ± SEM	Lower/Upper 95%CI	Mean ± SEM	Lower/Upper 95%CI	Mean ± SEM	Lower/Upper 95%CI	Mean ± SEM	Lower/Upper 95%CI
National	34,818	308.7 ± 3.5	301.7/315.6	86.0 ± 1.9	82.2/89.9	276.6 ± 3.8	269.2/284.0	64.6 ± 1.5	61.7/67.6
British Columbia	3616	357.4 ± 9.5^a^	338.7/376.1	89.0 ± 4.6	79.9/98.1	317.6 ± 9.0	300.0/335.3	77.9 ± 4.3	69.3/86.4
Prairies	9172	310.5 ± 5.8	299.1/321.9	88.6 ± 3.1	82.6/94.6	279.0 ± 6.2	266.8/291.3	66.1 ± 3.1	60.0/72.3
Ontario	10,837	308.2 ± 5.8	296.9/319.6	83.5 ± 2.5	78.5/88.5	274.8 ± 6.0	263.0/286.6	68.9 ± 2.6	63.8/74.0
Quebec	4746	291.3 ± 8.8	273.9/308.6	86.9 ± 6.0	75.1/98.7	263.8 ± 9.6	245.0/282.6	49.5 ± 3.0	43.6/55.5
Atlantic	6447	273.1 ± 5.9^b^	261.6/284.6	85.2 ± 4.4	76.5/93.9	246.8 ± 5.7	235.5/258.1	60.7 ± 2.7	55.3/66.0

^*^ Only those individuals who consumed some whole grain products were considered

# Only those individuals who consumed some refined grain products were considered

^ Only those individuals who consumed some seed products were considered

^a^indicates the significant highest level of consumption

^b^indicates the significant lowest level of consumption

*Whole Grains*: The number of Canadians consuming whole grain products ranged from 37% to 45%, the lowest being in the Atlantic region and the highest in British Columbia. British Columbia had the highest intake of whole grains and Ontario had the lowest (*p* < 0.05) (Table 1).

*Refined Grains*: The lowest prevalence of refined grain intake was found in the Atlantic region (75.8%) and the highest in Quebec (83.5%). However, in British Columbia the amount of refined grains consumed was significantly higher from all other regions (*p* < 0.05). The Atlantic region had the lowest consumption of refined grains, compared to the Prairies, and Ontario (*p* < 0.05) (Table 1).

*Seeds*: The prevalence of seed consumption was the lowest in the Atlantic region (32.7%) and the highest in British Columbia (42.2%). Similarly, the seed intake in grams per day was significantly higher in British Columbia compared to the national level and in the Atlantic and Quebec regions. Quebec had the lowest consumption of seeds, and it was significantly lower than the national level and the regions of British Columbia, Ontario and the Prairies (*p* < 0.05) (Table 1).

#### Grain category intake according to sex and age in different regions

**Fig. 1:**
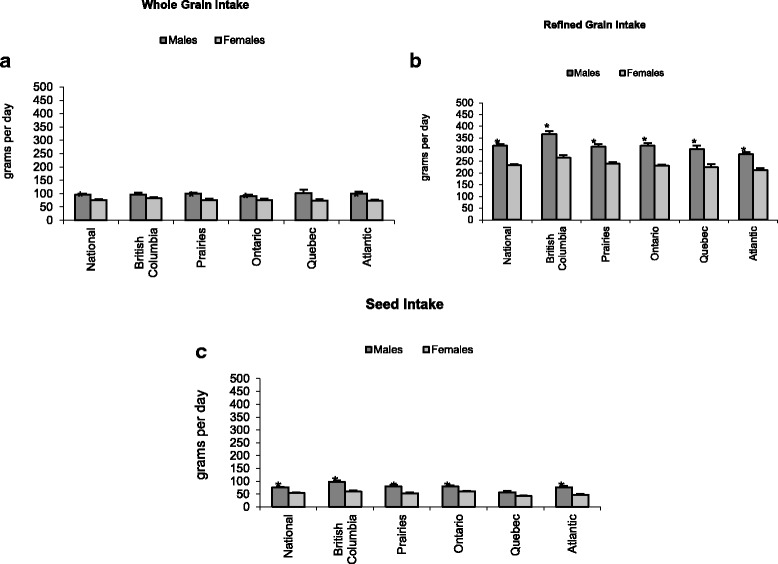
Categories of grain product intake in Canada according to sex (**a**) Whole Grain Intake, (**b**) Refined Grain Intake, (**c**) Seed Intake. * indicates significant sex difference at *p* < 0.05. Error bars represent the standard error of the estimate

*Whole Grains*: After correcting for total daily energy intake, males ate more whole grains (g/day), than females in all regions (*p* < 0.05), except in British Columbia and Quebec where differences between males and females were not significant (Fig. 1a). On the other hand, when comparing the prevalence instead of the consumption (g/day) of grain intake, a higher percentage of females consumed whole grains compared to males at the national level (43.2% vs. 38.8%) and in all regions, except in the Prairies where it was higher for males (43.7% vs. 40.6%) (Table 2). There were regional, as well as age and sex differences in consumption of the two common whole grains consumed in Canada: whole wheat bread and oatmeal. The national average intake of whole wheat bread was 76.0 g/day. Generally, men 19–30 and 31–50 years had the highest intake of whole wheat bread across the country (*p* < 0.05), with the exception of the Prairies where women 19–30 y had the highest intake. The national average intake of oatmeal was 110.0 g/day, with the highest consumption among men 31–50 y (142 g/day). However, regional differences showed that men over 50 years in the Prairies and Ontario had the highest consumption of oatmeal, 145 g/day and 150 g/day, respectively, while women 19–30 y in British Columbia had the highest intake (249 g/day). Men 31–50 y were the highest consumers of oatmeal in Quebec who consumed an average of 251 g/day. The highest consumption of oatmeal was among men 19-30y in the Atlantic Provinces who ate over three times the national average at 354 g/day.

**Table 2:** Categories of grain product intake in Canada by age and sex groups

Age/Sex Groups		Whole grains^a^		Refined grains^b^		Seeds^c^	
	N	Mean ± SEM	Lower/Upper 95%CI	Mean ± SEM	Lower/Upper 95%CI	Mean ± SEM	Lower/Upper 95%CI
Males	16,393	96.6 ± 3.3	90.1/103.0	317.6 ± 5.8	306.2/329.1	76.0 ± 2.5	71.1/80.9
Females	18,425	76.7 ± 2.1	72.7/80.8	234.0 ± 4.3	225.6/242.3	53.8 ± 1.5	50.7/56.8
under 8 y	5611	63.4 ± 2.8	58.0/68.9	196.7 ± 4.1	188.6/204.7	52.9 ± 2.6	47.8/58.1
9–18 M	4546	85.4 ± 3.2	79.2/91.7	332.7 ± 7.6	317.8/347.7	86.1 ± 4.2	77.9/94.3
9–18 F	4414	65.9 ± 2.4	61.1/70.6	271.5 ± 6.3	259.1/283.9	57.2 ± 2.2	53.0/61.5
19–30 M	1900	105.8 ± 7.6	90.9/120.6	394.5 ± 16.1	362.9/426.1	72.9 ± 5.6	61.8/84.0
19–30 F	2084	83.0 ± 7.4	68.5/97.6	290.0 ± 18.5	253.8/326.3	54.9 ± 3.7	47.6/62.1
31–50 M	2752	108.1 ± 8.6	91.3/124.9	355.7 ± 14.1	327.9/383.4	84.0 ± 5.6	72.9/95.0
31–50 F	2937	73.4 ± 4.4	64.7/82.1	244.3 ± 8.4	227.7/260.9	54.7 ± 3.1	48.6/60.8
Over 50 M	4335	94.6 ± 3.7	87.3/102.0	254.2 ± 6.6	241.1/267.2	70.8 ± 3.7	63.5/78.2
Over 50 F	6189	84.2 ± 3.3	77.6/90.8	189.5 ± 5.5	178.7/200.2	51.0 ± 3.2	44.7/57.3

^a^ Only those individuals who consumed some whole grain products were considered

^b^ Only those individuals who consumed some refined grain products were considered

^c^ Only those individuals who consumed some seed products were considered

*Refined Grains*: Males over 8y consumed significantly more refined grains (g/day) than females in all regions (*p* < 0.05) (Fig. 1b). Canadian males and females aged >50y had significantly lower intakes of refined grain products compared to other age groups over 8y (*p* < 0.05) (Table 2). Two of the most commonly consumed refined grain products were white bread and macaroni.

The mean intake of white bread in Canada was 56.0 g. Men 31–50 were the largest consumers of white bread, eating 69 g/day. Regionally, men 19–30 y in the Atlantic Provinces, British Columbia, and Quebec consumed the most white bread, 74.6 g/day, 82 g/day and 79 g/day respectively. Young men 9-18y in the Prairie Provinces were the highest consumers of white bread in that region at 74.5 g/day. This is distinct from Ontario where men 31–50 y were the top consumers of white bread in that province (67 g/day). A common Canadian enriched grain product is macaroni, and on average, Canadians consumed 291 g/day. Nationally, and in British Columbia, the Prairies, and Ontario men 19-30y were the top consumers of macaroni at 373 g/day, 399 g/day, 453 g/day, and 439 g/day respectively. Though, in Quebec and the Atlantic provinces, the younger people (men 9-18y) consumed the most macaroni at 379 g/day and 370 g/day, respectively.

*Seeds*: The consumption of seeds (g/day) was significantly higher for males when compared to females at the national and all regional levels, except in Quebec where the difference was not significant (Fig. 1c). At the national level, males aged 9-18y and >31y consumed more seeds than females (Table 2). Among the seed subcategories, the mean intake of beans was of 40.9 g/day at the national level. The highest consumption of beans was found in British Columbia, 54.7 g/day and the lowest in the Atlantic region, 26.6 g/day (*p* < 0.05). The intakes of beans at the national level and in British Columbia were significantly higher than in the Atlantic region. With the exception of Quebec, the intake of nuts in all regions was significantly higher in males compared to females. Men aged 19-30y and 31-50y were the highest consumers of nuts in British Columbia, the Prairies, Ontario and the Atlantic provinces. Young men in Quebec (9-18y) were the top consumers of nuts in that province, 62 g/day.

### Grain product intake and BMI in adults

#### Distribution of grain intake according to sex

**Fig. 2:**
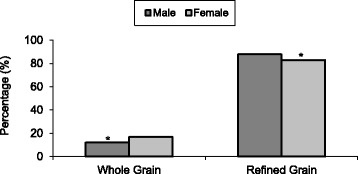
Distribution of adult whole grain and refined grain consumption according to sex. When intake >60%, consumers were categorized as either Whole Grain or Refined Grain consumers.* indicates significant sex difference according to category of grain intake (*p* < 0.001)

Considering only the adult population ≥ 19y, more than 80% of Canadian males and females were categorized as refined grain consumers. After dividing the total grain consumption into whole and refined grain intake categories, male versus female distributions under each category were 12% vs. 17% (*p* < 0.001) for whole grains, and 88% vs. 83% (*p* < 0.001) for refined grains (Fig. 2).

#### Characteristics of grain consumers according to sex

Female consumers showed significantly higher intakes of whole grains than males in lower, lower middle, and high income categories (Table 3). Also, within refined grain consumers, differences were observed between males and females according to income and physical activity (Table 3).

**Table 3:** Characteristics of whole grain versus refined grain consumers by sex

Consumers’ characteristics	Whole grain (%)		Refined grain (%)	
	Male	Female	Male	Female
Country of Origin				
Canadian born	43.7	56.3	51.0	49.0
Non-Canadian born	41.0	59.0	52.3	47.7
Household Income				
Lower	21.7	78.3^a^	41.7	58.3^a^
Lower middle	37.0	63.0^a^	49.8	50.2
Upper middle	50.4	49.6	53.3	46.7^a^
High	45.7	54.3^a^	54.8	45.2^a^
Food Security				
Food secure	41.3	58.7	52.1	47.9
Food insecure without hunger	40.8	59.2	48.6	51.4
Food insecure with hunger	38.5	61.5	44.4	55.6
Education				
Less than secondary	46.2	53.8	51.7	48.3
Secondary	35.4	65.6	47.5	52.5
Some post-secondary	43.9	56.1	53.9	46.1
Post-secondary	30.2	69.8	53.3	46.7
Physical Activity				
Active	46.6	53.4	59.0	41.0^a^
Moderate	36.9	63.1	53.5	46.5^a^
Inactive	41.6	58.4	49.2	50.8
BMI Classification				
Normal	30.1	69.9	51.2	48.9
Overweight	57.9	42.1	58.2	41.8
Obese	41.3	58.7	51.8	48.2

^a^ Significant sex difference at *p* < 0.05 (Pearson chi-square test)

#### Association between grain intake and BMI

Males were found to be at higher risk of being overweight or obese than females (adjusted odds ratio = 2.0, 95% CI = 1.03–3.89, *p* < 0.05). Also, in comparison to whole grains, consumption of refined grains was associated with higher trend for overweight and obesity (adjusted odds ratio = 1.93, 95% CI = 0.91–4.01, *p* = 0.08). Furthermore, in females, the odds of being overweight or obese were significantly higher for refined grain consumers (Adjusted odds ratio = 2.27, 95% CI = 1.01–5.12, *p* < 0.05), but this was no longer significant after adjusting for energy intake.

## Discussion

The national average grain intake was 309 g/day with the highest consumption of grains in British Columbia and the lowest in the Atlantic Provinces. Despite the recommended guidelines for whole grains being half the total servings of grains (6–7 serving/day), the actual consumption of whole-grain foods was very low compared to refined-grain foods. Traditional preferences for refined products, their taste and appearance, and unfamiliarity with appropriate cooking techniques are some of the barriers to increasing the consumption of whole-grain food [24, 25]. Considering that the mean intake of whole grains (86 g/day) was 3 times lower than that of refined grain products (277 g/day), there is a need for Canadians to increase their consumption of whole grains. In addition, approximately 80% of Canadians reported consuming 60% or more of their total grain intake as refined, which is not in agreement with Canada’s Food Guide recommendation that at least half of one’s total grain intake be from whole grains. Due to health benefits of whole grains, the current consumption of refined grains should be reduced to one-third or one-half of all grains, and be replaced by whole grains [11].

Canadians also consumed relatively low amounts of seeds at <65 g/day, or approximately 2 tablespoons per day. Canada’s Food Guide recommends individuals to “have meat alternatives such as beans, lentils or tofu often” and to “use dry roasted nuts and seeds…” [10]. There is evidence showing the potential health benefits of seeds including: whole grains, nuts and legumes, in decreasing the risks of cardiovascular disease and type 2 diabetes mellitus, two conditions which are highly prevalent in the country. Seeds consumption improves lipid profiles, glycemic responses and blood pressure, which are risk factors for cardiovascular disease and diabetes [26–28]. Therefore, encouraging increased consumption of seeds would be beneficial to the population, particularly in the Atlantic Provinces which showed the lowest intake of seeds.

Our findings are consistent with grain intake status in other countries. In the United Kingdom, whole-grain consumption has decreased over the last two decades to 14 g per day and 29% of adults report consuming no whole-grain foods [29, 30]. Similarly, in the United States, it is estimated that whole grains form less than 15% of the total grain consumption and only 6–8% of adults meet the target of three whole-grain servings per day [20, 31, 32]. In Australia, the National Nutrition Survey reported that almost 70% of the grains consumed are refined grains [33].

The general regional trend of a decreasing grain intake from West to East in Canada was found, yet there were important age and sex differences in consumption of grain products. While males aged 8-50y consumed more total and refined grains than females in all regions, men and women over 50y consumed significantly less grains (total and refined) than other age groups. Despite the higher amounts of whole grains consumed by males across the country, British Columbia and Quebec showed no significant difference in consumption rate between males and females. Furthermore, the consumption rate of whole grain products was only higher in 9-18y males when compared to females, thus suggesting that women may be more health conscious. This is evident by our results that physically active men consumed less whole grains when compared to women with similar activity levels, and that males (when compared to females) have an increased risk for being overweight/obese (adjusted odds ratio = 2.0, 95% CI = 1.03–3.89, *p* = 0.04). Grain food consumption might be different in accordance to ethnic or sex differences, however the contribution of refined grain foods exceeded that of whole grain products among the majority of ethnic or gender groups [34]. Consistent with our results, others have demonstrated using more whole-grains products by women rather than refined grains [35–37].

In our study, after adjusting for energy intake, women consuming refined grains showed an increasing trend in the risk for becoming overweight or obese, an association that is similar to the study by Ye et al. (2012) [38] who reported an inverse association between whole-grain intake and weight gain, with consistently less weight gain observed in those consuming 3–5 servings/day of whole grain compared with never consumers during 8–13 years of follow-up. In another study, women in the highest quintile of whole-grain and dietary fiber intake had a 23%, 49% lower risk of major weight gain, respectively [39]. Our finding of higher incidence of overweight and obesity from refined grain consumption compared to whole grains is consistent with results of others, showing that a high intake of refined grains leads to: higher BMI and WC; increased long-term weight gain; a higher fat mass; a higher BP; increased serum total and LDL cholesterol, fasting blood glucose, serum triglyceride; higher insulin resistance and diabetes prevalence [20, 40, 41, 30, 42–44]. It is notable that refined grains are a source of some important nutrients and also fortified with folate in Canada [9]. Our results indicate that the proportion of whole grain products consumption in total grain intake should be increased from 15% as we found in this study to higher level such as what is recommended by Health Canada as 50% of the total grain product intake [10].

Not all studies of grains consumption show body weight effects. Whole-grain bread consumption (60% rye, 40% wheat) in Finland was not associated with obesity in men [45], although the relationship was present in a smaller number of households. In Brazil, the prevalence of obesity was not related to the consumption of white rice, bread, biscuits, pasta and white flour in a large number of households [46]. American adults who consumed refined grains did not show any increase in BMI, percent body fat and trunk fat mass [21]. These discrepancies may be related to the different proportions of high-refined grain foods. While the details about foods included within the refined-grain category are not completely available, they usually consist of mixtures of low-fat grain foods and refined-grain foods, which are high in added fat, sugar, or sodium (e.g., pizza, doughnuts and cakes). Also, there are different confounding factors (including age, gender, ethnicity, economic status, physical activity, and underlying diseases) that might affect the association between obesity and grain intake. In addition, the popular Western diet, which is usually used for evaluating disease risk, has a high regional variation in the consumption and type of grain foods [11].

## Conclusion

In summary, age, sex and regional consumption differences were apparent in this study, leaving opportunities for encouraging consumption of whole grain products nationwide with an added focus on the male population, as well as on the Atlantic Provinces. Refined grains consumption was associated with an increase in body mass index in adults. These data suggest Canadians should be encouraged to reduce their current consumption of refined grains and to increase consumption of whole grains so that no more than one-half of all grains are refined, in order to meet the targets for whole-grain foods, which have proven beneficial health outcomes.

## Additional files

Additional file 1: Table S1. Grain products in three different categories. Description of data: Name of grain products in different categories. (DOCX 12 kb)

## Abbreviations

BMI: Body Mass Index
BP: Blood pressure
CCHS: Canadian Community Health Survey
CI: Confidence Interval
DRIs: Dietary Reference Intakes
WC: Waist circumference
