# Supplementary material for: National pattern of grain products consumption among Canadians in association with body weight status
Source: BMC Nutr. 2017 Aug 25;3:59. doi: 10.1186/s40795-017-0183-x (PMC7050848; doi:10.1186/s40795-017-0183-x)
Supplement: Supplementary file 1 — Grain products in three different categories. Description of data: Name of grain products in different categories. (DOCX 12 kb) [file 40795_2017_183_MOESM1_ESM.docx]

**Table S1** Grain products in three different categories

| **Whole Grains** | **Refined Grains** | **Seeds*** |
| --- | --- | --- |
| Whole wheat bread  Whole wheat pasta  Bulgur  Oatmeal  Popcorn  Whole grain cornmeal  Brown rice  Whole wheat buns  Whole wheat tortillas  Whole wheat crackers  Barley  Buckwheat  Rye  Whole grain breakfast cereals  Other grains: amaranth, millet, quinoa, sorghum, and triticale | White rice  White bread  Cornbread  Couscous  Flour tortillas  Noodles  Spaghetti  Pitas  Buns  Pretzels  White sandwich  Corn tortilla  Crackers  Pasta  Macaroni  Breakfast cereals | Beans (beans and pulses e.g. kidney beans, black beans)  Nuts (all types of nuts, nut butters and other foods that contain nuts such as trail mix)  Sesame seeds  Flaxseeds  Poppy seeds |

* Used in the second stage of the study that evaluated the association between grain intake and BMI; otherwise, seeds are considered as whole grain
